# Survey of non-conventional mental health care facilities in Côte d’Ivoire: first stage

**DOI:** 10.1186/s13033-021-00506-7

**Published:** 2021-11-24

**Authors:** Asseman Médard Koua, François Djo Bi Djo, Raymond N’Guessan Kouadio, Zoumana Coulibaly, Eric Sreu, Eméric Désiré Konandri, Aka N’zi Jean Vincent Koua, Sharon Huppertz, Gesine Heetderks, Michael Huppertz

**Affiliations:** 1 Department of Psychiatry, Université Alassane Ouattara, Bouaké, Côte d’Ivoire; 2 Department of Social Anthropology, Université Jean Lorougnon Guédé, Daloa, Côte d’Ivoire; 3 Department of Sociology, Université Peleforo Gon Coulibaly, Korhogo, Côte d’Ivoire; 4 Department of Geography, Université Alassane Ouattara, Bouaké, Côte d’Ivoire; 5 Department of Social Anthropology, Université Alassane Ouattara, Bouaké, Côte d’Ivoire; 6 Mindful Change Foundation, Darmstadt, Germany

**Keywords:** Global mental health, Non-conventional mental health care facilities, Prayer camp, Traditional healers, Côte d’Ivoire

## Abstract

**Background:**

Mental illnesses and disabilities as well as epileptic diseases remain an important public health issue. In Côte d’Ivoire, the provision of psychiatric care and specialised psychosocial facilities is almost non-existent. This study is based on the hypothesis that the care of people suffering from mental illness and epilepsy in Côte d’Ivoire is mainly in the hands of non-conventional mental health care facilities, including so-called ‘Prayer Camps’. These work according to traditional and spiritual principles and are mostly not registered or controlled by the Ivorian authorities.

**Methods:**

This study is the first stage of a multi-stage study. For the first stage, a quantitative method with an exploratory and descriptive aim was chosen. 541 non-conventional mental health care facilities in Côte d’Ivoire were mapped, typologised according to their spiritual orientation and treatment methods, and examined according to some charactersitics. Semi-structured interviews with 435 facility leaders were conducted.

**Results:**

The article provides a typology of four types of non-conventional mental health care facilities in Côte d’Ivoire including Christian Prayer Camps, Traditional Healing Centres, Phytotherapy Centres, and Roqya Centres. It explores their administrative embedding, the qualification of the facility leaders as well as their willingness, in principle, to cooperate with conventional mental health care centres. A considerable number of non-conventional expressed a desire or acceptance of cooperation with psychiatric organisations.

**Conclusions:**

The next stage of this multi-stage study will be to assess the clinical and legal situation of the patients in these centres. The aim is to interview the patients in order to analyse their perceptions and to capture the concerns of relatives and staff in the centres as well as the human rights situation in a mixed-method study. The long-term objective is to establish future cooperation between conventional psychiatric care providers and suitable non-conventional mental health care facilities and to implement a community mental health care policy in Côte d’Ivoire.

**Supplementary Information:**

The online version contains supplementary material available at 10.1186/s13033-021-00506-7.

## Background

Mental health, as well as cognitive and intellectual disabilities, remain a public health issue. In most Low- and Middle-Income Countries (LMICs), the provision of psychiatric care and specialised psychosocial facilities is almost non-existent in many countries. In some countries, there are however a few large, yet outdated, psychiatric hospitals. For families and patients, traditional or spiritual healers’ centres are often the first recourse for care. Human rights violations have been reported in both conventional and non-conventional mental health care settings to varying degrees in international surveys in many countries, documented mainly by investigative journalists and human rights organisations, but also in academic publications [1–8]. The situation of the mentally ill is particularly alarming in non-conventional mental health care facilities. In these facilities, patients are sometimes shackled and deprived of food and decent shelter. They may be physically and emotionally abused and deprived of their dignity. Health responses and legal provisions to protect the rights of these highly vulnerable populations are therefore essential.

It is in this context that the World Health Organization (WHO) has called for global and national mental health initiatives, including through the Quality Rights Program [9] and the Mental Health Action Plan 2013–2030 [10]. Initiatives such as the implementation of the Revised Mental Health Act 2012 in Ghana have enabled authorities to regulate abusive practices in Prayer Camps and thus free people with mental disorders who were previously shackled in these camps [11, 12], but profound changes are pending [1]. One of the priorities of the WHO Mental Health Action Plan [10] is the urgent need for increased availability of services that promote and protect human rights and dignity of people with mental illness [13]. As the Prayer Camps have provided care for the mentally ill so far, it is important to look closely at how they are doing this, in order to possibly advise the leaders on rights to quality care and encourage them to work with trained mental health professionals, provided that it is possible to include them—at least temporarily—in the development of community-based care [12, 13, 15, 16]. Emphasis is placed on the collaboration between mental health professionals and traditional and spiritual healers in the provision of quality care by drawing on examples from Ghana and Nigeria. Furthermore, this cooperation aims to monitor and supervise all conventional health care centres and service delivery points using the WHO QualityRights assessment tool.

In 2019 a regional survey was carried out in the Gbêkê region as a pilot study to report on the health situation of people with mental illness in spiritual healing centres, commonly known as ‘Prayer Camps’ [17]. This work was produced within the framework of a Community Mental Health project called the SAMENTACOM (Santé Mentale Communautaire) Project [18] in the Gbêkê region. This study made it possible to identify and map 71 Prayer Camps and to record around a hundred people with mental illness and epilepsy. The report also revealed violations of the fundamental human rights of patients in these facilities, characterised by deprivation of food, cases of forced fixation (shackling), and the inability of patients to access medical treatment [17].

There are real obstacles to working with those responsible for these informal community structures in light of silence within the legal community and indifference on the part of the administrative and health authorities. Therefore, it seemed appropriate to probe the full extent of the situation of people with mental illness and epilepsy in non-conventional mental health care facilities on a national level. Therefore, this first stage of a multi-stage study tried to find a relevant number of these non-conventional mental health care facilities caring for people with mental illness and epilepsy in Côte d’Ivoire with the aim of obtaining an overview of the supply of non-conventional mental health care. The aim was to record elementary characteristics of these facilities in order to be able to examine a selection that is as representative as possible in more detail in a second step. The long-term objective is to establish and implement future collaboration and a real community mental health care policy in Côte d’Ivoire.

Outline of the results presented in this text:

- An inventory and mapping of non-conventional mental health care facilities, also known as Prayer Camps, in parts of the Ivorian territory was started.
- A typology of 541 found non-conventional mental health care facilities was designed.
- Some structural characteristics of the non-conventional mental health care facilities were studied, such as the number of patients treated (inpatient and outpatient care), and the education and training of the facility leaders.
- The attitude of the facility leaders towards future collaboration with conventional psychiatric care was determined.

## Methods

In Côte d’Ivoire, the health authority does not maintain a database on non-conventional mental health care facilities. Data collection at regional or district level is also almost absent. Therefore, the data collection methodology of this study is composed of a pre-survey and the actual field survey. The pre-survey was carried out over one month by 31 community informants. The mission of 31 community informants was to build up a database of the existing range of non-conventional mental health care facilities. This was supported by the database of the National Traditional Medicine Promotion Program (PNPMT).

Once the database of non-conventional mental health care facilities in Côte d’Ivoire had been set up, 15 interviewers from four universities in Côte d’Ivoire (Bouaké, Korhogo, Daloa, and Abidjan) were recruited. They were trained in the use of the KoboCollect application, the steps to be taken once in the field, the objectives, methodology, schedule, and the expected issues of the survey.

After the recruitment and training of the interviewers, the field survey took place from 10 to 2020 to 2 July 2020 in the 31 regions of Côte d’Ivoire. There was an interruption of about seven weeks, as the COVID-19 pandemic interfered with the conduct of the study.

Based on the results of the pre-survey, the interviewers began their research in the regional capitals and their surrounding villages. Telephone arrangements with the heads of the facilities enabled the interviewers to flexibly plan their investigations and interviews in the individual regions. Public transport including bush taxis and motorbike taxis served as means of mobility for the interviewers in the field. The procedure was to use the bush taxis to travel to the villages. Once the non-conventional mental health care facility was identified, they borrowed motorbike taxis to visit the facility, which in some cases was located in another village, conduct interviews, and collect data. Activities were coordinated by the coordinator of the SAMENTACOM project (MCF-CI). Three researchers were in charge of supervising the field activities. To ensure ethical research, authorisations for data collection were obtained from the Director of the Psychiatric Hospital of Bouaké, the Ministry of Health and Public Hygiene, and the health authorities in every region.

For the survey, a quantitative method with an exploratory and descriptive aim was chosen. A standard semi-structured questionnaire was used for data collection (Additional file 1). This tool was embedded in 15 digital tablets equipped with GPS devices. The tablets enabled the interviewers to digitally record the responses of the facility managers to the questionnaire, as well as to collect ethnographic data, including photos.

The processing and analysis of the research data were carried out by a statistician. Data processing and analysis began with the creation of an online data backup platform to connect interviewers to the database during the surveys. This online database was created through the KoboToolBox application. The data collected by the interviewers were sent to the database. They were then processed and statistically analysed using IBM SPSS applications for multivariate processing and Microsoft Excel 2013. After the statistical processing, the data underwent a cartographic processing phase. The GPS coordinates of each structure were processed and transformed into geographical data using DNRS Gamin software. Then, the mapping of non-conventional mental health care facilities was carried out using the free software QGIS 2.18 in a WGS 1984 Coordinate and Reference System. Overall, the survey fulfilled its expectations, resulting in consistent data that has been processed and analysed.

## Results

The study found 541 non-conventional mental health care facilities. Non-conventional mental health care facilities refer to any private mental health care facility not organised by the state and not oriented towards science based medical psychiatry and neurology. However, 16.08 % of the facilities have a village authorisation, 14.78 % are recognised by their respectively associated higher-level structures and 13.86 % have a certificate from the Ministry of the Interior or the national program for the promotion of traditional medicine. There was no evidence of any kind of control exercised on the part of these institutions and organisations.

**Fig. 1:**
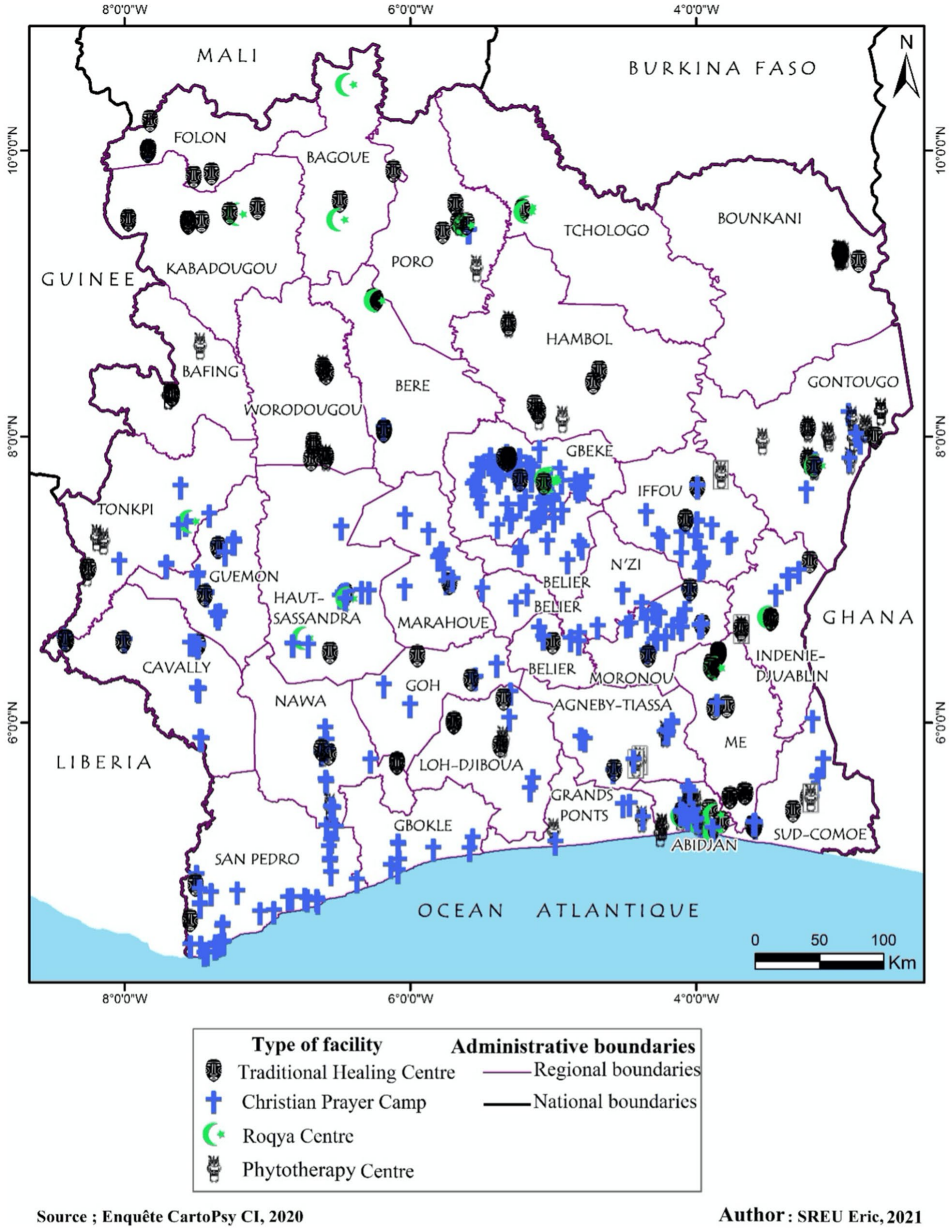
Map of regional distribution of surveyed non-conventional mental health care facilities by type of facility

Overall, the facilities surveyed in this study are mainly located in the Southern half of Côte d’Ivoire and are equally located in both rural and urban areas. The region of Gbêkê, including its capital Bouaké, represents 15.53 % of the surveyed facilities with a total of 84 facilities, making it the highest recorded number in the survey (Fig. 1).

Four different types of co-existing non-conventional mental health care facilities involved in the care of people with mental illness and epilepsy were identified. The categorisation of the non-conventional mental health care facilities implies a typification in which two aspects played a role: the spiritual orientation and the treatment practice. The four types include Christian Prayer Camps, Traditional Healing Centres, Muslim Roqya Centres, and Phytotherapy Centres. This categorisation results from the designations of the leaders of the surveyed facilities based on their specific practice and from the terminology of the National Traditional Medicine Promotion Program (PNPMT) (Table 1). The terms ‘facility’, ‘centre’, and ‘camp’ are used synonymously in this text and cover facilities of various sizes and frequencies of patients.

**Table 1:** Distribution of surveyed non-conventional mental health care facilities by type of facility

Type of Facility	Total Number	Percentage (%)
Christian Prayer Camp	326	60.26
Traditional Healing Centre	127	23.48
Phytotherapy Centre	59	10.91
Roqya Centre	29	5.36
Total	541	100

### Christian Prayer Camps

The data reveals that Christian Prayer Camps seem to be the most dominant non-conventional mental health care facility in Côte d’Ivoire. 326 Christian Prayer Camps have been recorded which account for 60.26 % of all surveyed facilities. These are under the responsibility of a Christian religious leader (pastor, prophet, priest), who is said to hold healing gifts received from God. They treat physical, spiritual, and mental health problems through prayer, fasting, and additional practices including medicinal plants. Sometimes they employ violent measures such as shackling and beating.

These Christian Prayer Camps are each associated with diverse Christian religious movements including the Christian Ministry Alliance (CMA), the Celestial Church of Christ, the Dehima Church, Pentecostal churches, and the Harriste Church. The CMA dominates the surveyed Prayer Camps by accounting for 25%. Looking at the 15 most frequented Christian Prayer Camps (out of 326 overall), the number of patients receiving treatment during the conduct of the survey ranges between 11 and 130. Accordingly, the facilities vary significantly in size.

It should be noted that these centres do not have a patient identification register. We could only determine these estimates on the basis of statements made by the leaders of the facilities. This also applies to the following data of the other facility types.

### Traditional healing centres

This study identified Traditional Healing Centres as the second most common non-conventional type of mental health care facility in Côte d’Ivoire. 127 recorded centres represent 23.48% of all 541 surveyed facilities and were found all over the country. A Traditional Healing Centre is defined by the fact that it treats health problems by combining local African spirituality with extracts from plants, trees, animal skin, and bones. Traditional healers, in addition to using local or traditional medicinal practices, in some instances resort to spiritual practices based on traditional religions. The combination of medicinal and spiritual treatment enables them to treat mental illness on both a physical and spiritual level.

There are many different affiliations among Traditional Healers. The recorded Traditional Healing Centres are dominated by healers who practice animist practices, which are employed in a total of 83% of the centres surveyed. This is followed by the Marabout (healers using Islamic and spiritual practice) who account for 6% of the centres, the Komian (spiritual priests or mediums in Akan culture) accounting for 8%, and diviners (healers who appeal to certain deities or ancestors) with 3%. Out of the 15 most frequented Traditional Healing Centres, the number of patients in care during the conduct of the survey ranges between 7 and 320.

### Phytotherapy centres

The third type of non-conventional mental health care facilities identified and investigated in this study are Phytotherapy Centres. 59 Phytotherapy Centres were recorded in this survey, accounting for 10.9% of the total number of surveyed facilities. These facilities are defined by the leadership of a Phytotherapist who makes use of plants and tree extracts as medicinal plants for the treatment of health problems. In the present context, there are Phytotherapists who have obtained recognition from the National Program for the Promotion of Traditional Medicine of the Côte d’Ivoire. Looking at the 5 most frequented Phytotherapy Centres, the number of patients receiving treatment at the time of the survey varies between 20 and 60.

### Roqya centres

With 29 recorded facilities, Roqya Centres account for 5.36% of all surveyed non-conventional mental health care facilities in this study. They are defined as Islamic facilities lead by a Muslim religious leader who treats occult diseases through Koranic verses and substances such as water, honey, herbs, oil, and perfume. The treatment is drawn and inspired from Koranic medicine, which often proceeds with exorcism sessions using techniques of atonement from the satanic spirit called Djinn or Sheitan, which is said to be the cause of all types of illness. In the 5 most frequented Roqya Centres identified, the number of patients receiving treatment at the time of the survey ranges between 10 and 120.

### Type of care and qualification of the facility leaders

Nearly half of the investigated facilities provide inpatient care and ambulatory care (46%). While 30% solely provide ambulatory care, 24% of the facilities offer only inpatient care. According to the facilities, the families are often involved in the care and nourishment of the patients. However, it frequently occurs that after having sent the patient to the facility, families abandon patients starting the first day or over time (for lack of means or as a conscious decision) and care is solely provided by the facility. In about half of the surveyed facilities, treatment is “free of charge” and based on donations from families based on successful treatment. In the remaining (50%) of the surveyed facilities, treatment is usually paid as a fixed fee. However, in both cases, any other costs relating to food, clothing, and other necessities that need to be paid for during the patients’ stay are not included.

Of the 541 surveyed non-conventional mental health care facilities, the leaders of 80.40% (435) of these facilities were interviewed. About 44% of those surveyed facility managers are unschooled and 26% have primary education. Those with secondary education make up 23% of the total, while 7% have tertiary education. In this environment, educational attainment has no relevance, as the mastery of healing techniques and gifts is paramount. However, the interviewed facility managers stated that they pursue associated professional activities or carry out secondary activities occasionally during the exercise of their function as facility managers, while the majority is engaged in agriculture (65%). 17% of those surveyed mainly engaged in housework. 12% stated that they are working as craftsmen and 3% reported to be working in the private sector or as public servants. Non-conventional mental health care facilities are predominantly male-dominated. Approximately 7% of the surveyed facility leaders were men, compared to 33% women. The presence of women in this sector is most visible in the Christian Prayer Camps and the Komian, as well as with Traditional Healers in Akan country, where the practice has always been dominated by women. The presence of women in charge of Prayer Camps is part of a Christian context, where in some communities women have the right and legitimacy to exercise a religious ministry.

### Attitude towards the care provided in psychiatric care services

The survey shows that the majority of facility leaders have an accepting attitude towards the care provided in psychiatric care services. Moreover, nearly half of the interviewees express a positive attitude towards cooperation with psychiatric care services. More precisely, 199 camp leaders considered psychiatric care provided in conventional psychiatric care facilities as ‘acceptable’, 96 leaders described it as ‘satisfactory’, 132 leaders did not have an opinion about it, and 11 considered it to be ‘bad’.

Overall, 248 camp leaders (57%) expressed their need for ‘knowledge sharing’ with psychiatric care services and 238 declared their ‘desire/need for assistance and training for better health care management/provision’. 205 interviewees spoke in favour of patient exchanges and 125 expressed the need for consultation within the facility. This is in line with the finding that 280 facility leaders suggested the inclusion of non-conventional mental health care facilities in the care of patients for better health care delivery. Beyond collaboration in terms of health treatment, the construction of sanitary structures (187) and the provision of equipment for existing facilities (156) were suggested measures for the improvement of the overall health care delivery. 128 camp leaders argued in favor of the integration of mental health care in primary health care centres.

## Discussion

The study attempts to gain a first impression of how prevalent non-conventional mental health care facilities are in West African countries. With limited resources and a small number of researchers, this study found 541 non-conventional mental health care facilities in Côte d’Ivoire. The survey started in Bouaké, in the centre of the country, but the researchers spread out to the other areas.

The question arises whether an estimate of the total number of Christian Prayer Camps, Traditional Healing Centres, Phytotherapy Centres, and Roqya Centres in Côte d’Ivoire is possible based on this survey. We can attempt a rough extrapolation: based on the approach taken in this survey, we can assume that most non-conventional mental health care facilities were identified in the Gbêke region, since Bouaké, the capital of this region, was the research team’s physical location and they are well connected in the surrounding area. It was therefore easiest for the team to logistically cover this region, which has a population of about 1 million. If we assume that all non-conventional mental health care facilities were identified in this region—there were 84—and that the number of non-conventional mental health care facilities in the whole country is about the same, and if we extrapolate this number to the total population of Côte d’Ivoire - about 24 million - we arrive at a total of 2016 centres in the entire country. Based on this estimate, the 541 centres recorded in this study make up about 26.8 % of the total number of centres running in the country were. However, both assumptions are questionable, as presumably not all facilities in Gbêke were recorded, so that the total number of non-conventional mental health care facilities in the country is probably even higher. Whether there are regional differences can only be clarified by a more comprehensive study, or if all non-conventional mental health care facilities were required to apply for a license for their activities and were registered, which would be a welcome improvement.

However, the number of facilities found is high enough to provide information about the different types of centres, the numbers of people with mental illness they treat, the educational or professional background of the facility leaders, and their attitudes towards possible cooperation with psychiatrically trained staff.

In the study, a considerable number of mental health care facility leaders expressed a desire or acceptance of cooperation with psychiatric organisations. However, this would need to go hand in hand with the outlawing of practices that mistreat and humiliate people with mental illness. This would require general training in WHO Quality Rights, as well as an agreement with leaders to observe them in their centres.

## Conclusions

The article provides a typology of four types of non-conventional mental health care facilities in Côte d’Ivoire according to their spiritual orientation and treatment methods including Christian Prayer Camps, Traditional Healing Centres, Phytotherapy Centres, and Roqya Centres. Through semi-structured interviews it provides results on the qualification of 435 facility leaders as well as their willingness, in principle, to cooperate with conventional mental health care centres. A considerable number of the leaders expressed a desire or acceptance of cooperation with psychiatric organisations. This data can be used by authorities and non-governmental organisations to support decision-making.

This first phase of a multi-stage study does not provide information on the social background of the leaders of the facilities, the therapeutic background of the patients, the living conditions of the people, the norms that govern the functioning of these facilities, and the belief systems related to mental illness and epilepsy in Côte d’Ivoire. The study did not take into account patients’ perceptions as well as perspectives of the patients’ relatives.

In the absence of data on the outcomes of treatments from the perspective of patients and families, it sounds premature to endorse an approach of future cooperation. However, cooperation between the Ivorian healthcare system and these centres seems to be a suitable option, especially given the lack of alternatives. The centres are predominantly the first points of contact for people with mental illness and epilepsy in the country who are mostly brought there by their families. This will not change easily. In the case the care in the non-conventional facilities is not replaced by an adequate, outpatient, psychiatrically competent mental health care system, constructive cooperation seems to us to be the next step. However, further, more detailed research and practical steps are needed to evaluate this perspective more precisely.

The study was planned as a multi-phase study. A second phase of the study is needed which will examine about 100 non-conventional mental health care facility leaders in more detail and request socio-demographic data on the individual patients and leaders, treatment methods, spiritual and ethical background. The investigators will stay in the centres and record their observations. In addition, cooperation between psychiatrically competent health care centres and a group of about 20 non-conventional mental health care facilities will be initiated and evaluated.

If cooperation could be established and combined with training for non-conventional mental health care facility leaders, there is hope that an urgently needed greater state provision of outpatient psychiatric care might well meet with a willingness on the part of the non-conventional facilities to make use of this help, especially if it can be integrated into the care provided in the centres.

## Supplementary Information

**Additional file 1**: Questionnaire. 

## Data Availability

The datasets used and/or analysed during the current study are available from the corresponding author on reasonable request.

## Abbreviations

LMICs: Low- and middle-income countries
WHO: World Health Organization
SAMENTACOM: Santé Mentale Communautaire
MCF-CI: The Mindful Change Foundation-Côte d’Ivoire
PNPMT: National Traditional Medicine Promotion Programme
CMA: Christian Ministry Alliance
