# Supplementary material for: Survey of non-conventional mental health care facilities in Côte d’Ivoire: first stage
Source: Int J Ment Health Syst. 2021 Nov 24;15:83. doi: 10.1186/s13033-021-00506-7 (PMC8611630; doi:10.1186/s13033-021-00506-7)
Supplement: Supplementary file 1 — Additional file 1: Questionnaire. [file 13033_2021_506_MOESM1_ESM.pdf]

CARTO PSY C.I 2020

Informations géographiques

Entrer la date et l'heure
Date d'enregistrement

yyyy-mm-dd   hh:mm

Le nom de l'enquêteur
Nom de l'Agent

- Agent 1
- Agent 2
- Agent 3
- Agent 4
- Agent 5
- Agent 6
- Agent 7
- Agent 8
- Agent 9
- Agent 10
- Agent 11
- Agent 12
- Agent 13
- Agent 14
- Agent 15

Dans quelle region vous trouvez vous?
Région

- Agneby Tiassa
- Abidjan district
- Bafing
- Bagoué
- Bélier
- Béré
- Bounkani
- Cavally
- Folon
- Gboklê
- Goh
- Gontougo
- Guémon
- Hambol
- Haut Sassandra
- Iffou
- Indenié Djuablin
- Kabadougou
- Grands Ponts
- Loh Djiboua
- Marahoué
- La Mé
- Nawa
- Nzi
- Poro
- San Pédro
- Sud comoé
- Tchologo
- Tonkpi
- Worodougou
- Folon

- ◯ Yamoussoukro District

Dans quelle localité êtes vous ou quelle est la localité la plus proche de vous?
ecrivez directement

non renseigné

Votre position Gps
actionnez la Position GPS du centre

non renseigné

latitude (x.y °)

longitude (x.y °)

altitude (m)

accuracy (m)

Typologie de la structure

Quel est le type de structure?
cochez une case

- ◯ Camp de guerisseur traditionnel
- ◯ Camp de prière chrétien
- ◯ Camp de prière musulman ou centre Roqya
- ◯ ONG oeuvrant pour la santé mentale
- ◯ Traditherapeute

Quel est le nom de votre structure?
ecrivez directement

En quelle année fut créée votre structure
écrivez l'année

Quel est le type architectural du centre?
cochez une case

- ◯ Habitat Villageois (en banco et paille)
- ◯ Habitat spontanné (fait avec materiaux de recuperation tels les baches et planches)
- ◯ Habitat moderne (en brique de ciment)
- ◯ Habitat mixte (moderne + villageois)

Quel est le nom du responsable de la structure?
ecrivez directement

A quel type d'église ou de ministère évangélique appartient votre structure (CMA, Celeste, UESSO, Reveil, Papa Nouveau, Dehima etc.......) ? si c'est un centre Roqya à quel type d'islam correspond t-il (Chiite, sunnite, zaydite, etc...) pour les autres religions vous pouvez aussi preciser
mentionnez directement la typologie de la religion ou le ministère auquel appartient la structure

Informations sur les responsables

Sexe ou Genre du responsable
cochez une case

- ◯ Masculin
- ◯ Feminin

Quel est le statut Matrimonial du responsable de la structure?
cochez une case

- ◯ Marié monogame
- ◯ Célibataire
- ◯ En concubinage
- ◯ marié polygame
- ◯ Veuf (ve)

De quelle ethnie êtes vous?
écrivez l'ethnie

Quel est le niveau d'instruction du responsable?
cochez une case

- ◯ Aucun
- ◯ Primaire
- ◯ Secondaire
- ◯ Supérieur

Quelles sont vos compétences professionnelles ou quel travail exerciez vous?
Renseignez directement

____

Possedez vous un document permettant d'exercer vos activités dans la légalité?
cochez une case

- ◯ Oui
- ◯ Non

Type d'autorisation obtenues
cochez une case

- ◯ Recepissé
- ◯ Agrément ou Numero arrêté
- ◯ Autorisation villageoise
- ◯ Aucun

Numéro du récépissé ou du numéro arrêté
écrivez le numéro du récépissé ou le numéro arrêté

____

Pratiques et mode de collaboration

Capacité d'accueil de la structure (lits)
ecrivez le nombre

____

Capacité d'accueil de la structure (Chambre ou maisonnette)
ecrivez le nombre

____

Quelles sont les maladies mentales que vous traitez au sein de votre structure?
cochez une ou plusieurs cases

- ☐ Epilepsie
- ☐ Folie ou maladie mentale
- ☐ Retard mental

Quel traitement médicamenteux offrez vous aux malades mentaux?
cochez une case

- ◯ Traitement avec psychotropes (médicaments en comprimés prescrits aux malades mentaux)
- ◯ Traitement traditionnel avec les plantes médicinales
- ◯ psychotropes + plantes traditionnelles
- ◯ Aucun

Quel traitement spirituel offrez vous aux malades mentaux?
cochez une case

- ◯ Christianisme (jeune, prière, eau benie et seances d'exorcisme)
- ◯ Islam (méthode roqya et autres)
- ◯ Réligion traditionnelle (komian, incantations, fétiche)
- ◯ Autres religions (Boudhisme, Eckankar, Rose croix etc.....)
- ◯ Aucun

Combien coûte le traitement pour une maladie mentale dans votre camp?

- ◯ gratuit
- ◯ moins de 5000 FCFA
- ◯ entre 5000 et 10 000 FCFA
- ◯ entre 11000 fr et 20 000 FCFA
- ◯ entre 21 000 et 50 000 FCFA
- ◯ plus de 50 000

Qui s'occupe de la prise en charge du malade mental dans votre structure?

- ◯ les parents du malade
- ◯ le malade lui même
- ◯ le responsable du camp ou de la structure

Type de suivi du malade mental
cochez une case

- ◯ Hospitalisation
- ◯ Ambulatoire
- ◯ Hospitalisation et ambulatoire

Travaillez vous en collaboration avec les autres structures de prise en charge des malades mentaux?
cochez une case

- ◯ Oui
- ◯ Non

Quel est votre avis sur la qualité des soins delivrés dans les autres structures de prise en charge des malades mentaux?

cochez une case

- ◯ Satisfaisant
- ◯ Acceptable
- ◯ Mauvais
- ◯ Aucun

Comment comptez vous collaborer avec les structures sanitaires pour la prise en charge des malades mentaux?

Cochez une ou plusieurs cases

- ☐ Aucune collaboration souhaitée
- ☐ Assistance et formation pour une meilleure prise en charge médicale
- ☐ Echange de malade
- ☐ Partage de connaissance
- ☐ Consultation au sein de votre structure

Quelles sont vos suggestions pour une meilleure prise en charge des malades mentaux ?

cochez une ou plusieurs cases

- ☐ Construction de structures sanitaires
- ☐ Equipement des structures existantes
- ☐ Integration des soins de santé mentale dans les centres de santé primaires
- ☐ participation des religieux et tradithérapeutres dans la prise en charge des malades

Combien de malades mentaux avez vous sur votre site?

saisir le nombre

____
